# Retention and promotion of women and underrepresented minority faculty in science and engineering at four large land grant institutions

**DOI:** 10.1371/journal.pone.0187285

**Published:** 2017-11-01

**Authors:** Marcia Gumpertz, Raifu Durodoye, Emily Griffith, Alyson Wilson

**Affiliations:** 1 Office for Institutional Equity and Diversity, North Carolina State University, Raleigh, North Carolina, United States of America; 2 Office of Institutional Research and Effectiveness, Virginia Tech, Blacksburg, Virginia, United States of America; 3 Department of Statistics, North Carolina State University, Raleigh, North Carolina, United States of America; Iowa State University, UNITED STATES

## Abstract

The current climate on college campuses has brought new urgency to the need to increase faculty diversity. In STEM fields particularly, the dearth of underrepresented minority (URM) and female faculty is severe. The retention and success of African American, Hispanic/Latino, American Indian and female faculty have direct implications for the quality and diversity of the future scientific workforce. Understanding the ways retention patterns differ by discipline and institution is crucial for developing a diverse faculty. This study investigates tenure attainment, retention, and time to promotion to full professor for women and URM faculty. We analyze personnel records for assistant and associate professors hired or appointed from 1992 to 2015 at four large land grant institutions. Representation of women and URM faculty in STEM disciplines increased substantially from 1992 to 2015, but mostly for women and Hispanic faculty and more slowly for black and American Indian faculty.

**Results by gender:**

In the most recent cohort, 2002–2015, the experiences of men and women differed substantially among STEM disciplines. Female assistant professors were more likely than men to leave the institution and to leave without tenure in engineering, but not in the agricultural, biological and biomedical sciences and natural resources or physical and mathematical sciences. In contrast, the median times to promotion from associate to full professor were similar for women and men in engineering and the physical and mathematical sciences, but one to two years longer for women than men in the agricultural, biological and biomedical sciences and natural resources.

**Results for underrepresented minority faculty:**

URM faculty hiring is increasing, but is well below the proportions earning doctoral degrees in STEM disciplines. The results are variable and because of the small numbers of URM faculty, the precision and power for comparing URM faculty to other faculty were low. In three of the four institutions, lower fractions of URM faculty than other faculty hired in the 2002–2006 time frame left without tenure. Also, in the biological and biomedical and physical and mathematical sciences no URM faculty left without tenure. On the other hand, at two of the institutions, significantly more URM faculty left before their tenth anniversary than other faculty and in engineering significantly more URM faculty than other faculty left before their tenth anniversary. We did not find significant differences in promotion patterns between URM and other faculty.

## Introduction

In Fall 2015, protests at about 60 college campuses across the U.S. brought new attention to demands and initiatives focused on increasing faculty diversity (e.g., [1–6]). The movement put a spotlight on the underrepresentation of historically marginalized groups and the lack of progress made to redress these issues. Women continue to be severely underrepresented among STEM (science, technology, engineering and mathematical science) faculty [7] and “faculty of color remain underrepresented and their achievements in the academy almost invisible” [8].

In recent years there has been great concern about the “revolving door for underrepresented minority faculty” [9–11], with evidence for the view that retention is at least as important as recruitment [12]. Moreno et al. [9] found that nearly three in five new URM faculty hired went to replace URM faculty who had left the institution. Concerning women, Thomas et al. [13] constructed stochastic models to demonstrate that faculty composition will never converge to parity unless recruiting, retention, and promotion to full professor are equal to men’s. Recruiting, retention, and career progression must all be increased in order to reverse under-representation of women and minority faculty in science and engineering faculties [14].

In 2012 Kaminski and Geisler [15] reported that retention rates and time to promotion for women faculty were similar to men in STEM disciplines. This differs from the many reports (e.g, [16,17]) of the leaky pipeline for women at all stages of an academic career. Kaminiski and Geisler did find that women leave the tenure track earlier than men in mathematics. Xu [18] demonstrated that faculty turnover can vary greatly among disciplines. Taylor and Froyd [19] estimated survival rates for women in engineering at Texas A&M almost 20 percentage points lower than for men. Box-Steffensmeier et al. [20], studying faculty in the social sciences, found no difference in retention between women and men, but did find that men have a higher chance of being promoted from assistant to associate professor than women. These reports raise two important questions: (1) how variable are results among different disciplines and institutions, and (2) how do retention and promotion rates for URM faculty compare to rates of other faculty?.

This study investigates faculty retention and time to promotion by race and by gender within STEM disciplines using institutional data for all tenure track assistant and associate professors hired in these disciplines at four large land grant universities categorized as highest research activity doctoral granting institutions. Land grant institutions employ a substantial fraction of all tenured and tenure track faculty in the U.S., 16% in 2015 [21].

## Materials and methods

### Data, definitions and outcomes

We use institutional data for all faculty hired into tenure track assistant professor positions in STEM disciplines at four institutions, labeled LG1-LG4 (2,077 individual faculty, Table 1), and all tenured and tenure-track faculty hired or promoted to associate professor appointments in STEM disciplines (1,793 individuals, Table 2). Previous studies [15,20][22], relied upon publicly-available CVs, websites and catalogs.

**Table 1:** Number of assistant professors appointed in STEM disciplines, by gender and by URM status for each institution. Percent of total faculty is in parentheses.

Institution	Year of Appointment as Tenure Track Assistant Professor									
	**1992–1996**		**1997–2001**		**2002–2006**		**2007–2011**		2012–2015*	
	**F**	**M**	**F**	**M**	**F**	**M**	**F**	**M**	**F**	**M**
**LG1**	19 (13%)	132	35 (24%)	113	45 (29%)	110	42 (28%)	109	30 (28%)	77
**LG2**	16 (37%)	27	22 (32%)	47	21 (31%)	46	37 (33%)	74	24 (32%)	50
**LG3**	NA		27 (23%)	89	38 (28%)	100	36 (29%)	90	41 (27%)	110
**LG4**	NA		NA		46 (23%)	151	41 (21%)	157	20 (27%)	55
	**1992–1996**		**1997–2001**		**2002–2006**		**2007–2011**		2011–2015*	
	**URM**	**Other**	**URM**	**Other**	**URM**	**Other**	**URM**	**Other**	**URM**	**Other**
**LG1**	10 (7%)	141	13 (9%)	135	11 (7%)	144	16 (11%)	135	10 (9%)	97
**LG2**	1 (2%)	42	3 (4%)	66	5 (7%)	62	11 (10%)	100	4 (5%)	70
**LG3**	NA		6 (5%)	110	13 (9%)	125	12 (10%)	114	11 (7%)	140
**LG4**	NA		NA		14 (7%)	183	22 (11%)	176	3 (4%)	72

*This cohort is for four years, faculty hired from 2012–2015.

**Table 2:** Number of faculty hired or promoted to associate professor in STEM disciplines by gender and by URM status for each institution.

Institution	Year of Appointment as Tenure Track Associate Professor										
	**1992–1996**		**1997–2001**		**2002–2006**		**2007–2011**			2012–1015*	
	**F**	**M**	**F**	**M**	**F**	**M**	**F**		**M**	**F**	**M**
**LG1**	25	127	14	108	24	106	39		110	46	83
**LG2**	1	12	14	41	20	40	26		59	28	64
**LG3**	NA		18	72	25	98	28		101	33	78
**LG4**	NA		NA		5	47	29		123	33	116
	**1992–1996**		**1997–2001**		**2002–2006**		**2007–2011**			**2012–2015**	
	**URM**	**Other**	**URM**	**Other**	**URM**	**Other**	**URM**	**Other**		**URM**	**Other**
**LG1**	7	145	7	115	12	118	10	139		16	113
**LG2**	0	13	6	49	1	59	5	80		9	83
**LG3**	NA		5	85	7	116	9	120		4	107
**LG4**	NA		NA		3	49	12	140		16	133

*This cohort is for four years, faculty hired or promoted to associate professor from 2012–2015.

We define STEM as all departments within the subfields of the agricultural sciences and natural resources, the biological and biomedical sciences, the physical and mathematical sciences, and engineering in line with the 2014 Survey of Earned Doctorates [22]. Black or African American, Hispanic/Latino and American Indian faculty are included in the underrepresented (URM) faculty group. The number of years of available data varied among the institutions; time to promotion/tenure or time to exit were available for each faculty member hired or appointed from 1992–2015 for LG1 and LG2, 1997–2015 for LG3, and 2002–2015 for LG4.

For faculty hired from 2002–2015, we report three outcomes for each institution and for each discipline group (agricultural sciences and natural resources, biological and biomedical sciences, physical and mathematical sciences, and engineering) by gender and race/ethnicity group. The outcomes are

1. Proportion of assistant professors exiting without tenure,
2. Survival functions of those originally hired as assistant professors,
3. Time to promotion from associate to full professor.

In selected cases, where earlier cohorts are available, we compute the outcomes for earlier cohorts and report on trends over time.

### Incidence of leaving without tenure

For assistant professors appointed from 2002 to the present, cumulative incidence curves [23] were constructed to estimate the probability of exiting the university without tenure. Cumulative incidence curves provide summary information on survival in the presence of competing risks. There are two ways that an assistant professor might leave the assistant professor rank: (1) by leaving the institution while still a pre-tenure assistant professor, or (2) by obtaining tenure and promotion to associate professor. These two possibilities are mutually exclusive outcomes; one precludes the other. In the language of survival analysis, these two outcomes are competing risks. The event of interest in this study is the event of leaving the university while still an assistant professor, before taking up a tenured associate professor position. Faculty who were still pre-tenure assistant professors in 2015, the last year for which data were available, or had died while an assistant professor were considered censored. Cumulative incidence curves for men and women and for URM vs other faculty were compared using Gray’s test [24]. The cumulative incidence curves were fitted separately for each institution; however, if the differences between demographic groups showed similar patterns for all four institutions, the four institutions’ tests for differences by gender and race/ethnicity were combined by stratification. Because the numbers of URM faculty are small, the statistical tests do not have much power.

Note that it is not possible to distinguish between faculty who did not apply for tenure, those who applied but were not granted tenure and those who were granted tenure in the summer but opted to leave the institution before the new rank took effect the following fall semester; all of these cases appear in the data as faculty who left without tenure.

### Faculty retention

Life table survival estimates for time until exiting the university at any rank were computed for all faculty who were appointed as tenure track assistant professor positions at each institution separately by gender and by URM status. Wilcoxon tests were used to compare survival functions for different groups.

### Time to promotion from associate professor to professor

Cumulative incidence curves [23] were constructed to estimate the probability of promotion from associate to full professor according to time in rank. In this case, the competing risks are (1) promotion to full professor, and (2) leaving the institution while still an associate professor. The event of interest was the time to promotion to full professor, the competing event is exiting the institution while an associate professor, and faculty who remained at the university but had not been promoted by 2015 or who died while serving as associate professor were considered censored. Gray’s test [24] was used to compare cumulative incidence curves for men and women and for URM vs other faculty within an institution.

### Ethics statement

Approval for the study protocol and use of the data was obtained from the Institutional Review Boards of the two institutions involved in conducting this research. The full names of the institutional review boards of the two institutions are (1) the Institutional Review Board for the Protection of Human Subjects in Research (IRB) and (2) the Institution Review Board (IRB). Two additional institutions provided de-identified data. One of these institutions provided a letter from their Research Integrity and Compliance Review Office stating that their “staff are not considered engaged in this research. It was determined that providing these (deidentified secondary) data does not meet the regulatory definition of research…” and the fourth institution’s Human Research Protection Program provided a letter authorizing us to “conduct your research recruiting in the method described”. Informed consent was not requested because we used institutional data that is maintained for usual university business, including assessing demographic hiring and retention trends. North Carolina General Statutes do not specify race and sex as “confidential” personnel information, nor are race and sex specified as data that are “open to inspection”. Race and sex data are voluntarily provided by employees (with the option not to provide). Demographic information collected on employees is typically reported in aggregate to avoid potential problems with perception/reality of inappropriate use of the information. All other information in the dataset (name, tenure status, rank, college, department, age, and email address) are public information. To protect confidentiality, our IRB agreement states “The reports and manuscripts that we plan to produce will contain estimated survival curves, numbers of faculty used to compute the estimates, and median survival time and risk estimates; they will not contain any data on individual faculty or on small groups of faculty of fewer than 5 people.”

### Notes on definitions of URM faculty

Race and ethnicity data pose several difficulties. Definitions and racial/ethnic categories have changed over time and the data can be prone to errors. We used the most recent race and ethnicity recorded for each individual. At two of the institutions, information on specific races and ethnicities were not available for faculty who identified as multiracial or who identified with two or more racial/ethnic groups. For these institutions, multiracial faculty were not included in the URM group because many are Asian/White or other non-URM faculty. URM status was determined for all faculty regardless of citizenship or visa status. However, at one of the institutions, race and ethnicity were not reported for international faculty, so international faculty were not considered URM for that institution.

## Results

### Hiring demographics over time

Appointments to tenure track assistant professor positions in STEM disciplines generally increased for underrepresented minorities over time, reaching 10 to 11 percent at the four institutions in 2007–2011, but declining in the most recent cohort. The gains in hiring differed among ethnic and racial groups (Table 3). Most of the increases came as a result of hiring Hispanic/Latino faculty. Black faculty constituted no more than 2% of tenure track assistant professors hired from 2002–2015 at three of the four institutions studied. American Indian faculty hiring was less than 1% at all of the institutions. Hispanic hiring increased substantially across cohorts, making up 5–6% of tenure track assistant professor hires from 2002–2015.

**Table 3:** Percent URM* of tenure track assistant professors hired.

		1992–1996	1997–2001	2002–2015 †
**LG1**	**Black**	5%	3%	4%
	**Hispanic**	1%	6%	5%
	**American Indian**	1%	0	1%
**LG2**	**Black**	0	1%	1%
	**Hispanic**	2%	3%	6%
	**American Indian**	0	0	1%
**LG3**	**Black**		2%	2%
	**Hispanic**		3%	6%
	**American Indian**		0	0.2%
**LG4**	**Black**			2%
	**Hispanic**			6%
	**American Indian**			0.2%

*Note that faculty may identify with more than one racial/ethnic category, so the totals may be larger than the percent of URM faculty reported in Table 1.

^†^ Note that this is a period of 14 years, whereas the first two columns are cohorts of 5 years each.

The proportion of tenure track assistant professor positions that went to women increased over time at three of the four institutions, reaching 27–28% in the most recent time period. At LG2 the female share of appointments was substantially higher in all time periods, ranging from 32% to 37% over the entire 1997–2015 time frame. At that institution, women are more highly represented in the biological and agricultural sciences than at other institutions, but there are very few women in engineering (Table 4).

**Table 4:** Distribution of women across disciplines*.

Institution	Number of Assistant Professors	Agriculture and Natural Resources	Biological and Biomedical Sciences	Engineering	Physical and Math Sciences
**LG1**	Women: 117	32 (27%)	26 (22%)	22 (19%)	37 (32%)
	All: 413	101 (24%)	79 (17%)	124 (30%)	118 (29%)
**LG2**	Women:82	29 (35%)	23 (28%)	8 (10%)	22 (27%)
	All: 252	76 (30%)	52 (21%)	47 (19%)	77 (31%)
**LG3**	Women: 115	31 (27%)	24 (21%)	36 (31%)	24 (21%)
	All: 415	110 (27%)	63 (15%)	148 (36%)	94 (23%)
**LG4**	Women: 107	17 (16%)	14 (13%)	39 (36%)	37 (35%)
	All: 470	79 (17%)	51 (11%)	194 (41%)	146 (31%)

*Number of tenured and tenure track assistant professors hired from 2002–2015, with percent of faculty in each discipline group in parentheses. Percentages total 100% across each row.

### Women’s probability of leaving without tenure

At all four institutions, the final probability of leaving without tenure was similar for men and women in the 2002–2015 cohort (Table 5) when all STEM disciplines are pooled together, although the shape of the cumulative incidence curves for men and women were significantly different at one of the institutions (LG4, p-value = .09), with women leaving earlier than men, particularly in the 5^th^ year. In contrast, the fraction of women who left without tenure in the 1997–2001 cohort was much higher than that of men. At the three institutions for which we have data for faculty hired between 1997 and 2001, 37%, 36% and 44% of those female assistant professors left without tenure, while 21%, 28% and 22%, respectively, of male assistant professors left without tenure.

**Table 5:** Probability of exiting without tenure, by gender*.

	N	Percent Female	Prob(Exit without Tenure)** 95% confidence intervals in parentheses.		Compare Cumulative Incidence Curves for Men and Women
			Women	Men	P-Value***
**LG1**	413	28%	24% (15%–34%)	24% (19%–30%)	.97
**LG2**	252	33%	18% (10%–29%)	17% (10%–26%)	.34
**LG3**	415	28%	24% (15%–34%)	29% (22%–36%)	.99
**LG4**	470	23%	30% (21%–40%)	28% (22%–34%)	.09

* Assistant professors hired in STEM from 2002 to 2015.

** Estimated from cumulative incidence curves.

*** Gray’s test for equality of cumulative incidence functions.

**Fig 1:**
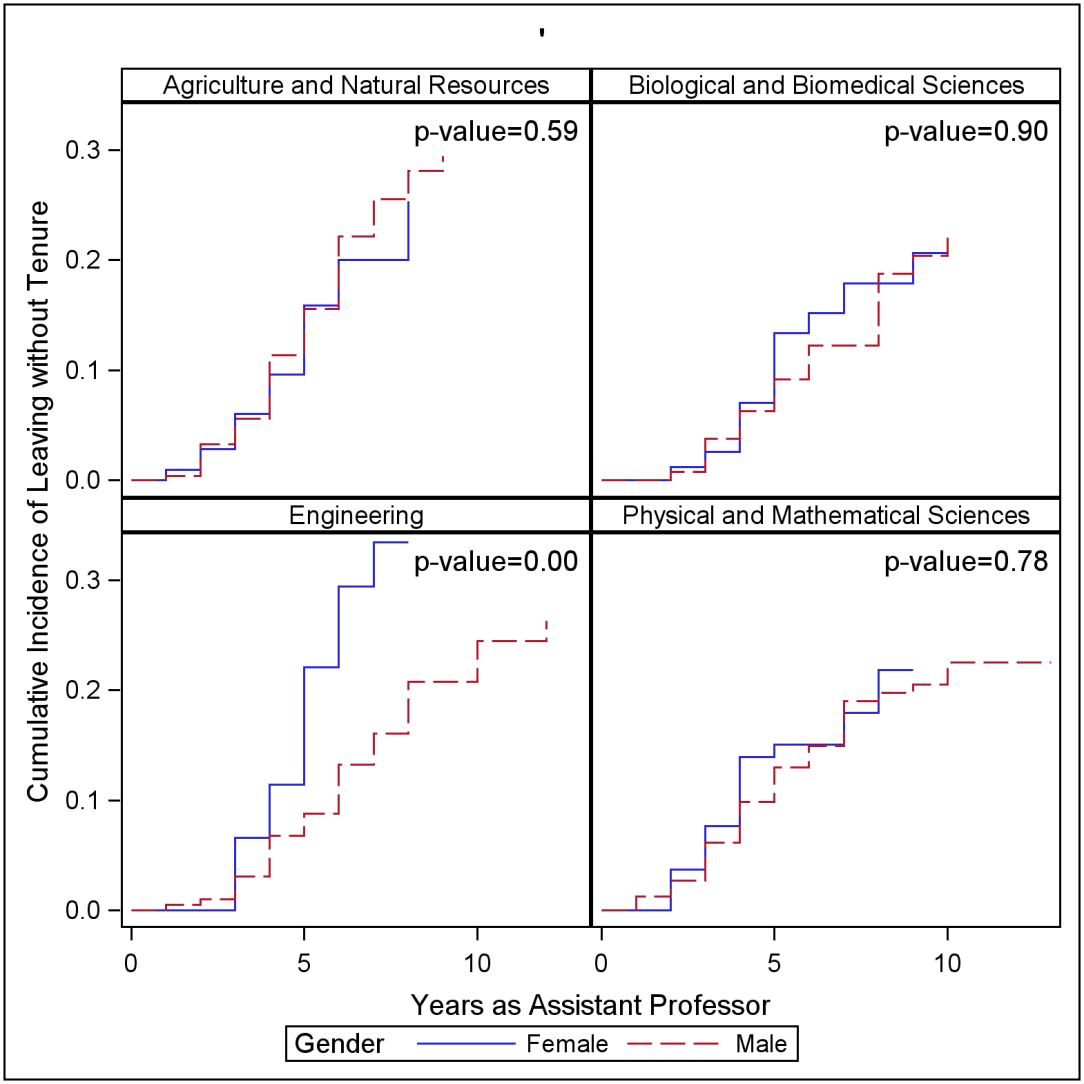
Cumulative incidence of leaving without tenure by discipline. Cumulative incidence curve for leaving without tenure, by discipline group, all four institutions combined, 2002–2015. The p-values indicate the significance of Gray’s test comparing the cumulative incidence curves for men and women.

Disaggregating the data by discipline reveals differences among disciplines in the size of gap, or lack of a gap, between men and women (Fig 1). In Engineering, women were more likely to leave without tenure than men (Fig 1). The estimated probability of leaving without tenure for women in Engineering is 33%, but for men is 26%. The difference in incidence of leaving is largest in year five; in year five 11% of women are estimated to leave, compared with 2% of men. We do not see differences between women and men in the other three groups of disciplines (Fig 1).

### Probability of URM faculty exiting prior to tenure

In the 2002–2015 cohort, URM faculty were *less* likely to leave without tenure than other faculty at three of the four institutions (Table 6), though these differences were not statistically significant. At LG3, however, URM faculty were more likely (50% vs 25%) to leave without tenure than other faculty, again not statistically significant. It is important to note that the numbers of URM faculty that have been hired and that have been in rank long enough to have been considered for promotion to associate professor are very small in these disciplines at all four institutions, ranging from 20 to 39 faculty hired over the 14-year period. Hence, the estimated probability of leaving for URM faculty is not very precise and the confidence intervals are wide. Among URM faculty hired at LG3 from 2002–2015, eight faculty actually left without tenure, 11 faculty were granted tenure, and 17 URM faculty were hired too recently to have been considered for tenure yet (Table 7). Looking at the previous cohort further demonstrates how difficult it is to make statements about patterns based on small numbers. In the 1997–2001 cohort all six assistant professors hired stayed on as associate professors with tenure at LG3. Data collected for this study do not support findings on whether climate at LG3 changed or whether differences are due solely to individual circumstances for faculty in the two cohorts.

**Table 6:** Cumulative incidence of exiting without tenure, by URM status and institution*.

	N	Number URM	Prob(Exit without Tenure) 95% confidence intervals in parentheses.	
			URM	Other
**LG1**	413	37	10% (2%–24%)	26% (21%–31%)
**LG2**	252	20	6% (0.3%–23%)	18% (12%–24%)
**LG3**	415	36	50% (26%–69%)	25% (20%–31%)
**LG4**	470	39	15% (5%–30%)	29% (23%–34%)

* Estimated from cumulative incidence curves of assistant professors hired from 2002 to 2015.

**Table 7:** Percent of underrepresented minority faculty who left the institution without tenure.

Institution	1992–1996	1997–2001	2002–2015*
**LG1**	30% (n = 10)	15% (n = 13)	10% (<5:20:14)
**LG2**	(n = 1)	33% (n = 3)	6% (<5:14:5)
**LG3**		0 (n = 6)	50% (8:11:17)
**LG4**			15% (5:25:9)

* The 2002–2015 cohort percentages are estimated from cumulative incidence curves. In parentheses are the number that left without tenure, the number granted tenure, and the number censored (e.g., still there but not yet tenured), separated by colons.

In the biological and biomedical sciences and in the physical and mathematical sciences, *no* URM assistant professors hired in the 2002–2015 time frame left without tenure (Table 8). No significant differences in the proportion leaving without tenure were seen between URM and other faculty in engineering or in the agricultural sciences and natural resources.

**Table 8:** Cumulative incidence of exiting without tenure, by URM status and discipline*.

	N	Number URM	Prob(Exit without Tenure) 95% confidence intervals in parentheses.	
			URM	Other
**Biological and Biomed Sci**	236	25	0	24% (17%–32%)
**Engineering**	513	49	44% (27%–59%)	27% (21%–33%)
**Agric Sci and Nat Resources**	366	38	23% (8%–42%)	28% (22%–35%)
**Physical and Math Sciences**	435	20	0	23% (18%–28%)

* Estimated from cumulative incidence curves of assistant professors hired from 2002 to 2015.

### Length of service of women

We focus now on total length of service rather than probability of achieving tenure. Kaminski and Geisler [15] reported a median time to departure of 10.9 years for assistant professors hired between 1990 and 2002 in a subset of STEM disciplines at 14 institutions. At the four institutions in our study, retention times were much longer than those reported by Kaminsky and Geisler for most categories of faculty. To compare, we used our first two cohorts, covering faculty hired from 1992–2001. During this time period data were available for three institutions. Two institutions submitted data covering the period of 1992–2001, with the third institution’s coverage periods spanning 1997–2001. Using these time periods, the median time to departure for women was 19 years at one institution and 11 years at another. At the third institution, 55% of female faculty were estimated to stay longer than 23 years, which was as far as our dataset extended. The median time to departure could not be estimated for men at any of the three institutions, meaning that a majority of men stayed at the institution longer than the number of years of available data, which in this case ranged from 14 to 23 years.

**Fig 2:**
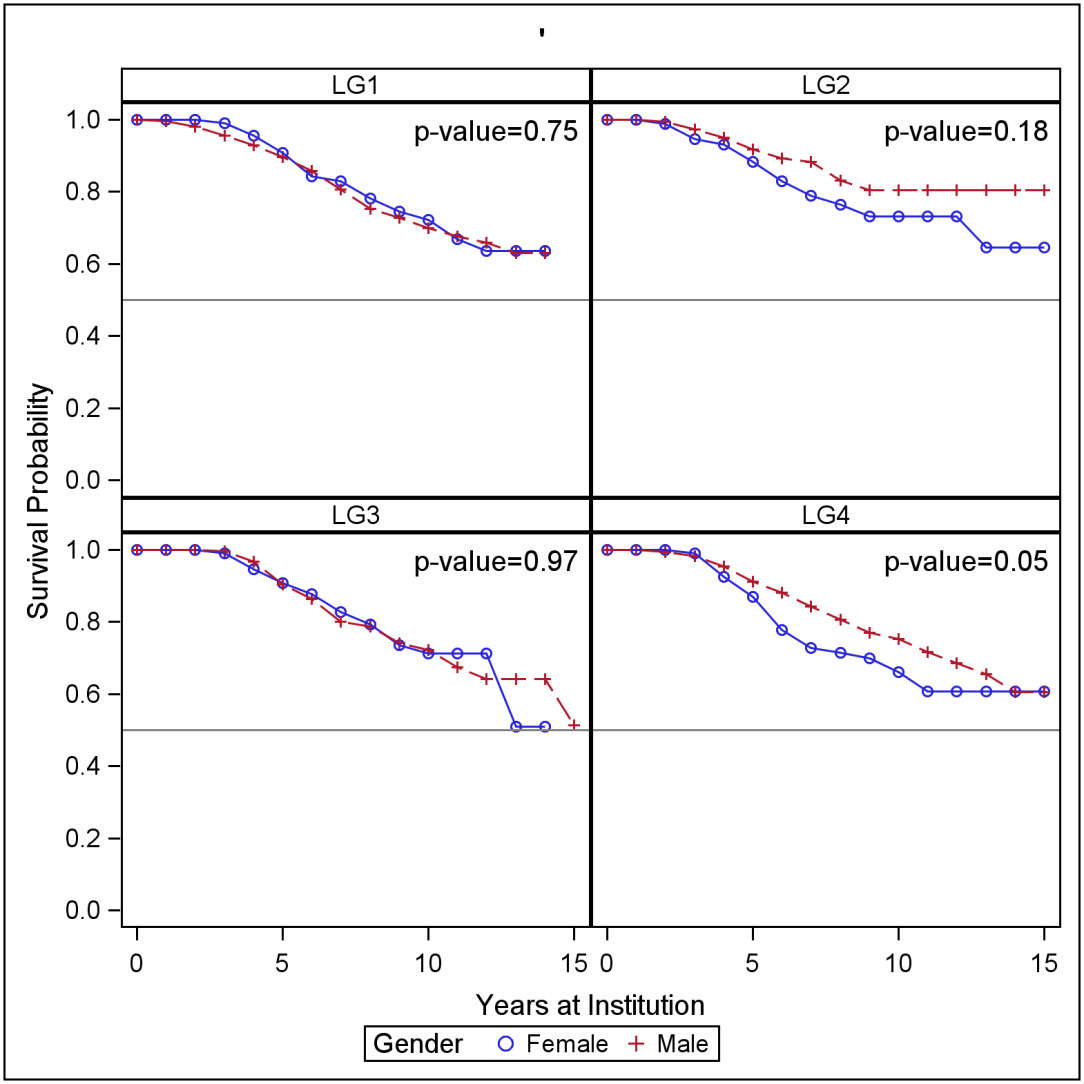
Probability of retention by institution and gender. Life table survival curves for tenure track assistant professors hired between 2002 and 2015. P-value indicates significance level of Wilcoxon test of equality of the life table estimates of survival for men and women.

**Fig 3:**
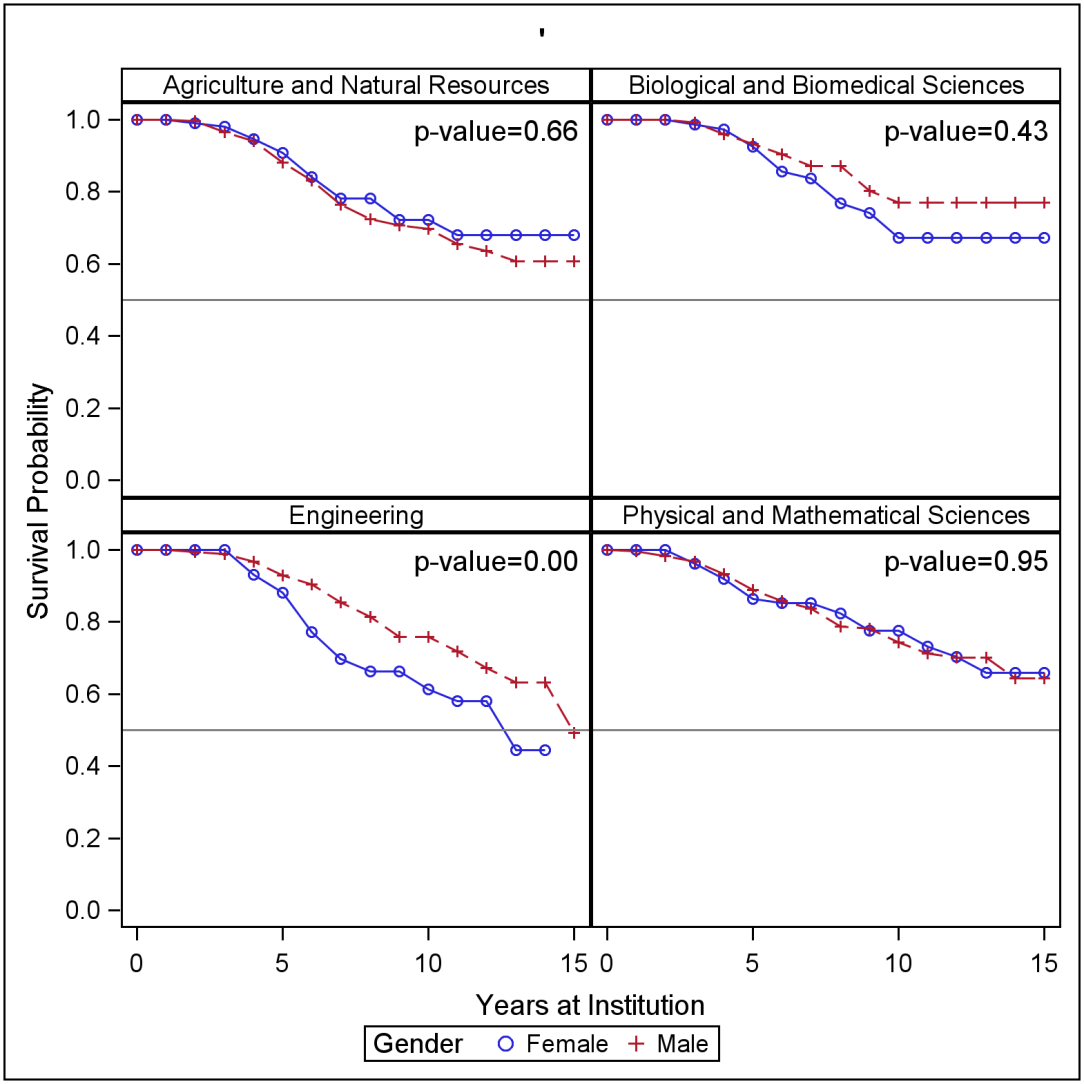
Probability of retention by discipline and gender. Life table estimates of probability of survival (retention), by discipline group and gender, for all institutions combined. P-values indicate the level of significance of the Wilcoxon test of equality of the curves for men and women.

We use two sets of cohorts to compare faculty retention across gender and race/ethnicity. The 2002–2006 cohort, which is available for all four institutions, provides a snapshot of the proportions of faculty who have stayed at the institution for ten years or more. The 2002–2015 cohorts provide additional information on the behavior of recently hired faculty who have joined the institution less than ten years ago. Female faculty longevity varies by institution and discipline. At LG1, LG2 and LG3 we see no differences in retention patterns between women and men (Fig 2 and Table 9). At LG4, a higher fraction of women are estimated to leave the institution within 10 years than men. In Engineering, women are at higher risk of leaving than men, particularly in years 3 through 7 (Fig 3). The years of highest risk for women are years 3, 5 and 6, in which 7%, 12% and 10% of women were estimated to depart.

**Table 9:** Number of faculty who exited before their 10th anniversary of hire, by gender, estimated two ways*.

	LG1		LG2		LG3		LG4	
	F	M	F	M	F	M	F	M
Exited within 10 yr, 2002–2006 cohort*	22% (10/45)	28% (31/110)	24% (5/21)	20% (9/46)	29% (11/38)	28% (28/100)	37% (17/46)	23% (34/151)
	p-value** = .68		p-value = .75		p-value = 1.0		p-value = .10	
Prob (exit within 10 yr)***	25%	28%	27%	20%	26%	26%	30%	23%

* Proportion of tenure track assistant professors hired from 2002–2006 who exited before the 10^th^ anniversary. Number exiting / Number hired is in parentheses.

** Fisher’s exact test for equal proportions, comparing women and men.

*** Probability of exiting before the 10^th^ anniversary is estimated from life table analysis of tenure track assistant professors hired between 2002 and 2015.

### Length of service of URM faculty

**Fig 4:**
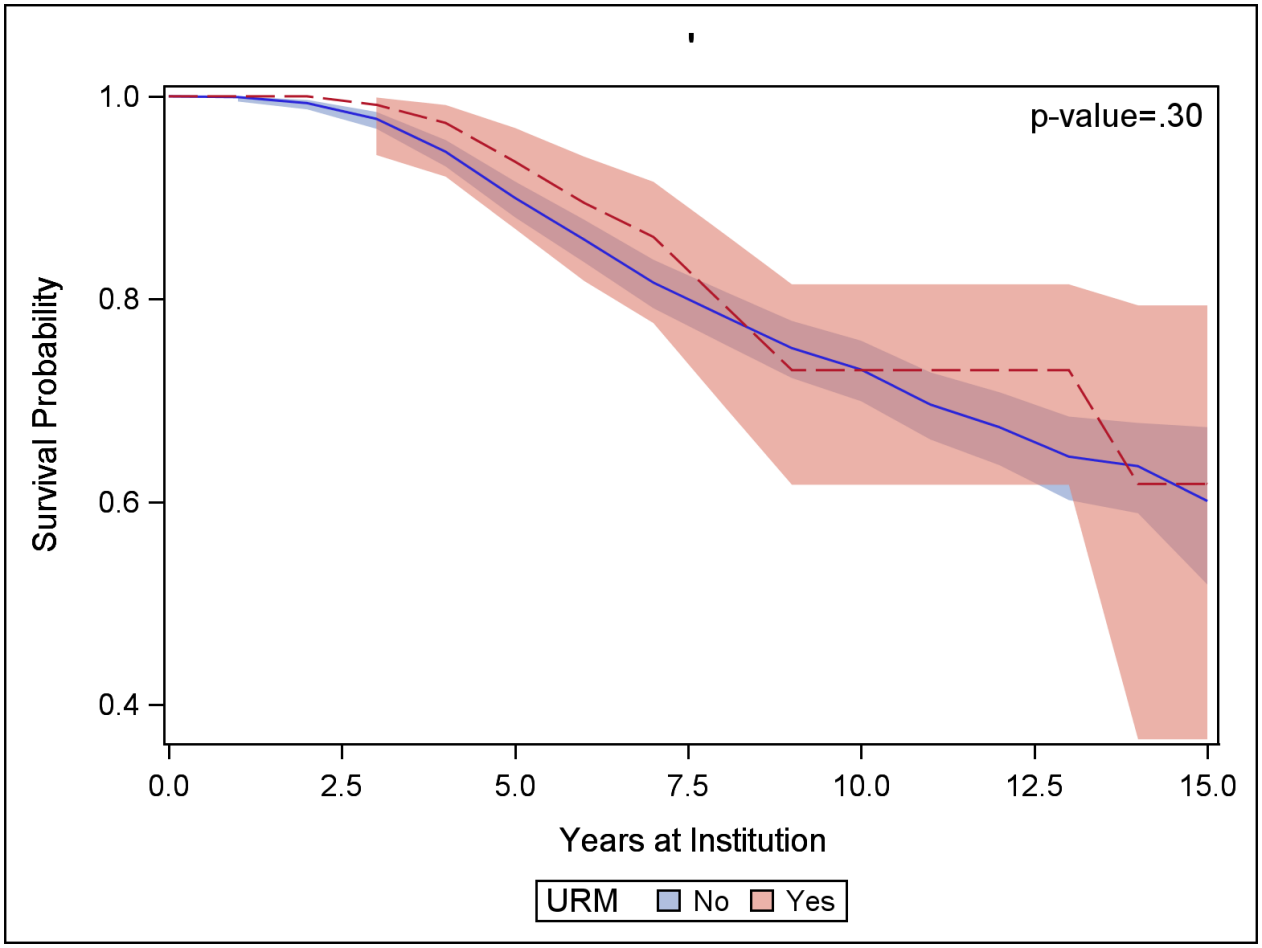
Probability of retention for URM faculty vs other faculty. Life table estimates of probability of survival, by URM status, with 95% confidence intervals. The p-value indicates the level of significance of the Wilcoxon test of equality of the curves for URM faculty and other faculty.

When all institutions and STEM disciplines are combined, we see no difference in retention patterns between URM faculty and other faculty (Fig 4). The number of URM faculty, 132 assistant professors, is large enough to obtain fairly precise estimates of retention up to about 5 years. The 95% confidence limits for probability of retention for 5 years or more are 87% to 97% (compared to the confidence limits of 88% to 92% for other faculty, which is based on 1418 faculty). The confidence intervals are wider for longer retention times. By 9 years, the width of the 95% confidence interval for URM faculty retention has widened to 19 percentage points: 62% to 81%, because only 35 URM faculty were hired more than 9 years ago and are still in the pool for estimating the survival probability.

When we look at individual institutions, however, the results of comparisons between populations differ among institutions. At both LG4 and LG1, URM faculty had *lower* incidence and estimated probability of leaving within 10 years than other faculty, but the differences are not statistically significant (Table 10). The other two institutions provide a contrast. In both LG2 and LG3 more URM faculty have left within 10 years than have stayed 10 years or more; 60% of URM faculty from LG2 and 54% of URM faculty from LG3 hired from 2002–2006 left within ten years; whereas only 18% and 26%, respectively, of other faculty departed in less than ten years (p-values = .06 and .14 for LG2 and LG3, respectively).

**Table 10:** Percentage of faculty who exited before their 10^th^ anniversary of hire, by URM status, estimated two ways.

	LG1		LG2		LG3		LG4	
	URM	Other	URM	Other	URM	Other	URM	Other
Exited within 10 yr, 2002–2006 cohort*	18% (2/11)	27% (39/144)	60% (3/5)	18% (11/62)	54% (7/13)	26% (32/125)	14% (2/14)	27% (49/183)
	p-value** = .75		p-value** = .06		p-value** = .14		p-value** = .56	
Prob (exit within 10 yr)***	15%	28%	40%	20%	41%	25%	15%	26%

*Proportion of tenure track assistant professors hired from 2002–2006 who exited before the 10^th^ anniversary. Number exiting / Number hired is in parentheses.

** Fisher’s exact test for equal proportions, comparing URM to other faculty.

*** Probability of exiting before the 10^th^ anniversary estimated from life table analysis of tenure track assistant professors hired between 2002 and 2015.

It is notable that *all* URM faculty hired in the 2002–2006 time frame in the biological and biomedical sciences and the physical and mathematical sciences stayed at their institutions ten years or more (Table 11). On the other hand, in engineering almost twice as high a fraction of URM faculty as other faculty exited within ten years. Larger numbers of URM faculty would be needed to come to more general conclusions about whether length of service differs between URM faculty and other faculty within specific disciplines.

**Table 11:** Percentage of faculty who exited before their 10^th^ anniversary of hire, by URM status and discipline, estimated two ways.

	Biological and Biomedical Sciences		Engineering		Agric Sciences and Natural Resources		Physical and Mathematical Sciences	
	URM	Other	URM	Other	URM	Other	URM	Other
Exited within 10 yr, 2002–2006 cohort*	0 (0/6)	26% (20/77)	47% (9/19)	25% (39/159)	45% (5/11)	29% (32/112)	0 (0/7)	24% (40/166)
	p-value** = .33		p-value** = .11		p-value** = .37		p-value** = .35	
Prob (exit within 10 yr)***	7%	24%	36%	24%	34%	29%	7%	23%

*Proportion of tenure track assistant professors hired from 2002–2006 who exited before the 10^th^ anniversary. Number exiting / Number hired is in parentheses.

**Fisher’s exact test for equal proportions, comparing URM to other faculty.

*** Probability of exiting before the 10^th^ anniversary estimated from life table analysis of tenure track assistant professors hired between 2002 and 2015.

### Time to promotion from associate to full professor for women

The patterns of promotion from associate to full professor differ significantly among the four groups of disciplines (p-value = .007, Table 12). Differences among institutions were not as pronounced, but still noticeable (p-value = .13). Over 70% of associate professors are promoted to full professor in the biological and biomedical sciences and the physical and mathematical sciences, compared with 58% in the agricultural sciences and natural resources. Within all of the disciplines except engineering, women are estimated to be less likely to be promoted in four years than men (Table 12). In the biological, biomedical, agricultural sciences and natural resources women are less likely to be promoted in 6 or 8 years than men. The median time to promotion for women is more than a year longer than for men in these disciplines.

**Table 12:** Estimated cumulative incidence of promotion to full professor, by discipline and gender*.

Years in Rank as Associate Professor	Agric Sci and Nat Resources		Biological and Biomed Sci		Engineering		Physical and Math Sciences	
	Women	Men	Women	Men	Women	Men	Women	Men
**4**	3%	6%	4%	14%	14%	11%	16%	21%
**6**	25%	36%	27%	46%	47%	38%	41%	45%
**8**	39%	50%	49%	57%	50%	51%	55%	56%
**Final**	56%	58%	65%	76%	56%	60%	74%	70%
**Median time to promotion**	10.0 yr	8.0 yr	8.2 yr	6.9 yr	8 yr	7.7 yr	6.9 yr	6.8 yr
CIC curves equal** **for four disciplines?** p-value = .007								
**CIC curves equal for four institutions?** p-value-.13								
**CIC curves equal for men and women within discipline?** p-value = .13 (stratified by discipline)								
**CIC curves equal for men and women within institution?** p-value = .13 (stratified by discipline)								

* Each row shows the estimated percentage of faculty promoted to full professor at or before the given number of years in rank. Faculty hired or promoted to tenure track or tenured associate professor rank from 2002 to 2015.

** Gray’s test for equality of CIC curves.

### Time to promotion from associate to full professor for URM faculty

We did not find any significant differences in the probability or time to promotion from associate to full professor between URM and other faculty, even when differences among disciplines or institutions are accounted for (Table 13).

**Table 13:** Estimated cumulative incidence of promotion to full professor, by discipline and race/ethnicity *.

Years in Rank as Associate Professor	Agric Sciences and Natural Resources		Biological and Biomedical Sciences		Engineering		Physical and Mathematical Sciences	
	URM N = 29	Other N = 293	URM N = 20	Other N = 188	URM N = 33	Other N = 424	URM N = 22	Other N = 352
**4**	6%	5%	9%	11%	8%	12%	12%	20%
**6**	21%	34%	43%	38%	41%	39%	30%	45%
**8**	52%	47%	60%	53%	70%	50%	63%	55%
**Final**	52%	59%	60%	72%	70%	58%	63%	70%
CIC curves equal** **for URM and other faculty within discipline?** p-value = .83 (stratified by discipline)								
**CIC curves equal for URM and other faculty—all disciplines combined?** p-value = .90								
**CIC curves equal for URM and other faculty within institutions?** p-value = .81 (stratified by institution)								

* Faculty hired or promoted to tenure track or tenured associate professor from 2002 to 2015.

** Gray’s test for equality of CIC curves

## Summary and discussion

Similar to previous reports [15], when all institutions and STEM disciplines are combined, few differences between men and women are seen in any of the promotion and retention outcomes: the incidence of exiting the university without tenure, overall length of service, or time to promotion from associate to full professor. The introduction posed the question of how variable results are among different disciplines and institutions. We found that faculty retention and time to promotion for women vary substantially among disciplines. Female engineering faculty who started as assistant professors had higher incidence of leaving than men, particularly in years 3 through 7, and left without tenure more frequently than men. We did not see this discrepancy in the other disciplines. On the other hand, time to promotion from associate to full professor averages one to two years longer for women than for men in the biological, biomedical and agricultural sciences and natural resources, but no differences in time to promotion by gender appear in the physical and mathematical sciences and engineering.

The four institutions in this study are very similar by several measures; they are all classified as highest research activity doctoral granting land grant institutions. Yet even among this homogeneous set of institutions and disciplines within the sciences and engineering, there is some variability among institutions, though not as large in magnitude as the differences among disciplines. At LG4, but not at the other institutions, female assistant professors were not retained at the institution as long as men. Institutions or discipline groups wishing to understand the experiences of women in their fields or institutions and to draw conclusions about needs to be met or action steps to take, should be aware of these differences and make discipline-specific or institution-specific estimates.

The second question posed in the introduction was how retention and promotion rates for URM faculty compare to rates for other faculty. Although the focus of this paper is faculty retention rather than hiring, the low number of URM faculty at the four institutions under study affects the conclusions that we are able to draw and our understanding about how to address issues of representation in higher education.

The small numbers prevent us from making conclusions about retention for African American/black, Hispanic/Latino, or American Indian faculty specifically, but the hiring data show some striking differences among racial/ethnic groups. Black or African American faculty have not been hired in as large proportions as earn doctorates, but Hispanic and American Indian faculty are being hired in approximately the same proportions as earn Ph.D.s in STEM disciplines. Further studies are needed to understand differences in hiring patterns, promotion and retention for specific underrepresented racial/ethnic groups.

Notably, *all* URM faculty hired as assistant professors from 2002–2015 in the biological and biomedical sciences or the physical and mathematical sciences earned tenure at their institutions. Because of the small numbers of URM faculty and resulting low power, no statistically significant differences were found in the incidence of leaving without tenure between URM faculty and other faculty at any institution, though at three of the four institutions smaller fractions of URM faculty left without tenure than other faculty. The picture for overall retention of URM faculty who started as assistant professors is more mixed. At two of the institutions in our study, significantly higher fractions of URM faculty than other faculty left the institution within ten years of hire. The biological, biomedical, physical and mathematical sciences are again notable: all URM faculty in these disciplines stayed at their institutions ten years or more. At the associate professor stage, no large differences in time to promotion from associate to full professor were discernable.

Understanding the specific patterns within a discipline or an institution is important for those wishing to evaluate or address disparities. It is also important to understand the large impact that departures of a few URM STEM faculty can have in an institution or discipline where the community is already very small. Our general conclusion is that URM faculty representation is so low that it limits our understanding of faculty retention and success. We did not have the statistical power to discern many differences and did not find consistent differences in career progression between URM faculty and other faculty. We found that in some disciplines URM faculty are retained at higher rates than other faculty, but also found some cases of specific institutions where URM retention is lower than other faculty.
